# Tube Wells

**Published:** 1877-12

**Authors:** 


### *TUBE WELLS.*

### OUR PATENT--FREE TO EVERYBODY.

The best well, and the one least liable to get out of order, is made by welding an iron point into one end of an iron pipe which is one and a half inches in diameter.

The point should not be more than four inches long, and rather blunt. It should be hardened, so that it will penetrate and break the stones that it meets when being driven down. About one hundred holes, a quarter of an inch in diameter, should be drilled through the two feet of the pipe, just above the point, to admit the water. The pipe may be in lengths of five or six feet, which can be coupled as it is driven. To drive down the pipe one man holds a wooden beetle on the upper end of pipe, whileanother strikes the beetle with a heavy sledge hammer. Tie a small piece of metal to the end of a string, and drop down the pipe to ascertain whether the water has been reached. The pipe should be driven four to six feet into the vein after the water is obtained. When the proper depth is reached you have a permanent, open well, into which no surface water can enter. Into this well insert an inch pipe (to which a pump is attached) to within one foot of bottom of well, pump out the sand and dirt and the work is completed. A little cement may be put into the space between the pipes, at the top, to prevent the possibility of surface dirt or water entering the well. A well made in this way steers clear of all pretended patents, and is really better than any other. We constructed a well of this description, assisted only by a man who used the sledge-hammer. Green's patent covers. "Wells made air-tight by attaching a pump to the lining of the well." Now, the lining of our well is the pipe we drive down, and to which *we do not attach a pump.* It simply makes a small, open well, into which we insert a pipe, with pump attached, as in any other well. For family use, a well one-and-a-quarter inches in diameter, into which a three-quarter inch pipe is inserted, and to which a three-quarter inch pump is attached, will supply an abundance of water for twenty families. A coupling for an inch pipe is one-and-five-eighth inches, outside diameter, and must be turned down one-fifth of an inch before it will enter an inch-and-a-half well. Gas pipes are in lengths of fifteen feet, so that not more than one coupling would be necessary, unless in wells where greater depth would require a different pump. Galvanized pipe should always be used for tube wells.

